# The Capacity of Generic Musculoskeletal Simulations to Predict Knee Joint Loading Using the CAMS-Knee Datasets

**DOI:** 10.1007/s10439-020-02465-5

**Published:** 2020-01-30

**Authors:** Zohreh Imani Nejad, Khalil Khalili, Seyyed Hamed Hosseini Nasab, Pascal Schütz, Philipp Damm, Adam Trepczynski, William R. Taylor, Colin R. Smith

**Affiliations:** 1 Department of Mechanical Engineering, University of Birjand, Birjand, Iran; 2 Institute for Biomechanics, ETH Zurich, Leopold-Ruzicka-Weg 4, 8093 Zurich, Switzerland; 3 Julius Wolff Institute, Charité – Universitätsmedizin Berlin, corporate member of Freie Universität Berlin, Humboldt-Universität zu Berlin, and Berlin Institute of Health, Berlin, Germany

**Keywords:** Instrumented knee implants, EMG, OpenSim, Knee contact force, Musculoskeletal modeling, CAMS-knee, Level walking, Squat

## Abstract

Musculoskeletal models enable non-invasive estimation of knee contact forces (KCFs) during functional movements. However, the redundant nature of the musculoskeletal system and uncertainty in model parameters necessitates that model predictions are critically evaluated. This study compared KCF and muscle activation patterns predicted using a scaled generic model and OpenSim static optimization tool against *in vivo* measurements from six patients in the CAMS-knee datasets during level walking and squatting. Generally, the total KCFs were under-predicted (RMS: 47.55%BW, *R*^2^: 0.92) throughout the gait cycle, but substiantially over-predicted (RMS: 105.7%BW, *R*^2^: 0.81) during squatting. To understand the underlying etiology of the errors, muscle activations were compared to electromyography (EMG) signals, and showed good agreement during level walking. For squatting, however, the muscle activations showed large descrepancies especially for the biceps femoris long head. Errors in the predicted KCF and muscle activation patterns were greatest during deep squat. Hence suggesting that the errors mainly originate from muscle represented at the hip and an associated muscle co-contraction at the knee. Furthermore, there were substaintial differences in the ranking of subjects and activities based on peak KCFs in the simulations versus measurements. Thus, future simulation study designs must account for subject-specific uncertainties in musculoskeletal predictions.

**Electronic supplementary material:**

The online version of this article (10.1007/s10439-020-02465-5) contains supplementary material, which is available to authorized users.

## Introduction

Knowledge of the internal musculoskeletal forces acting on the knee joint during dynamic functional movements has significant potential for informing injury and degenerative disease prevention strategies,^48^ improving the outcomes of orthopedic treatments,^31^ enhancing implant designs,^3^,^24^ and validating computational model predictions.^18^,^38^ Since the 1970s, multibody musculoskeletal models of increasing complexity have been proposed to predict such internal knee joint loading conditions.^45^ Currently, several musculoskeletal modeling software packages such as AnyBody,^7^ LifeModeler,^25^ SIMM,^9^ BodyMech,^13^ and OpenSim,^8^ provide simulation tools for predicting joint loading. However, while standard motion analysis measurements and rigid body mechanics can directly determine inter-segmental joint loads and moments, distribution of these loads to muscles, ligaments, and articular contact surfaces remains complicated by the inherent redundancy within the musculoskeletal system,^11^ particularily with regards to muscle co-contraction.^44^ Thus, *in vivo* validation remains a major obstacle in widespread acceptance of model predictions of knee loading and hence limits clinical translation of the technology.

Historically, EMG measurements of muscle activity have provided the primary validation methodology.^26^ The development of instrumented joint replacements introduced a new “gold standard” for model validation,^16^ and a number of these datasets have been made publicly available. Here, the Orthoload team have released datasets including knee contact forces, whole body marker kinematics, and ground reaction forces (GRFs) for a single subject performing a single trial of level walking (https://orthoload.com/comprehensive-data-sample/). In addition, the “Grand Challenge to Predict In Vivo Knee Loads” has released a comprehensive dataset including joint contact forces, marker kinematics, GRFs, EMG, computed tomography (CT) scans, and stationary fluoroscopy for four subjects perfoming normal and modified walking (https://simtk.org/projects/kneeloads).^12^,^19^

These publicly available benchmark validation datasets provide an invaluable resource for critically evaluating musculoskeletal modeling predictions. The Grand Challenge to Predict In Vivo Knee Loads competition inspired substantial improvements in the sophistication of modeling techniques, however the accuracy of the KCF predictions over the five years of the competition remained relatively unimproved.^19^ Here, the format of the Grand Challenge competition focused on evaluating the capacity of musculoskeletal models to predict absolute metrics of subject-specific KCFs during various forms of walking. However, the capacity of musculoskeletal models to predict KCFs during other activities has been less thoroughly studied.^37^,^43^ Furthermore, because musculoskeletal models have previously struggled to predict absolute measures of joint loading, they are often used instead to predict differences in metrics between e.g., subjects, groups of subjects, activities, or pathologies. However, due to the limited number of subjects and activities in the publicly available benchmark validation datasets, the ability to establish differences has not been extensively validated.

More recently, the “Comprehensive Assessment of the Musculoskeletal System” (CAMS-Knee) project released *in vivo* measured KCFs, skeletal knee joint kinematics using moving fluoroscopy, EMG, motion capture, and GRFs (https://cams-knee.orthoload.com/).^42^ These datasets now offer the opportunity to compare trial repetitions for multiple subjects, each performing five complete cycles of different activities of daily living. The objective of this study was therefore to use the CAMS-Knee datasets to evaluate the predictive capacity of generic open source (OpenSim) models to estimate knee joint loading throughout complete cycles of functional movements. This is intended to provide a baseline validation using the most standard tools available that can later be used to benchmark more complex models and simulation techniques.

## Materials and Methods

In this study, a validation of the absolute knee joint contact forces and moments, as well as muscle activations, was performed by comparing *in vivo* measurements versus modeling predictions during level walking and squatting. In addition, to establish whether subject-specific modelling predictions meaningfully represent real-world scenarios, a relative validation of the order of measured *versus* predicted KCFs for the different subjects and activities was performed.

### CAMS-Knee Datasets

The experimental data used in the current study were obtained from the CAMS-Knee datasets.^42^ The datasets include six patients (5 m, 1 f, age 68 ± 5 years, mass 88 ± 12 kg, height 173 ± 4 cm) with each possessing a cemented INNEX knee implant (Zimmer, Switzerland; FIXUC), for which the tibial component was instrumented to allow the measurement of six load components (three forces and three moments of tibio-femoral joint).^14^ Here, whole body kinematics were measured using 75 skin markers and a 26 camera motion capture system (Vicon, UMG, UK) at 100 Hz. GRFs were collected at 2000 Hz with six force plates embedded in the walkway (Kistler Instrumentation, Winterthur, Switzerland). Bilateral muscle activity for eight major lower limb muscles (rectus femoris, vastus medialis, vastus lateralis, tibialis anterior, semitendinosus, biceps femoris long head, medial gastrocnemius, and lateral gastrocnemius) were detected using a 16-channel wireless EMG system (Trigno, Delsys, USA) with signal delay of 48 ms. For each subject, five trials of level overground walking and squatting were simulated in this study. A cycle of level walking was defined as heel strike to heel strike of the instrumented leg. For squatting, a complete cycle was defined from upright standing to deep flexion back to upright standing.

### Musculoskeletal Modeling

**Figure 1:**
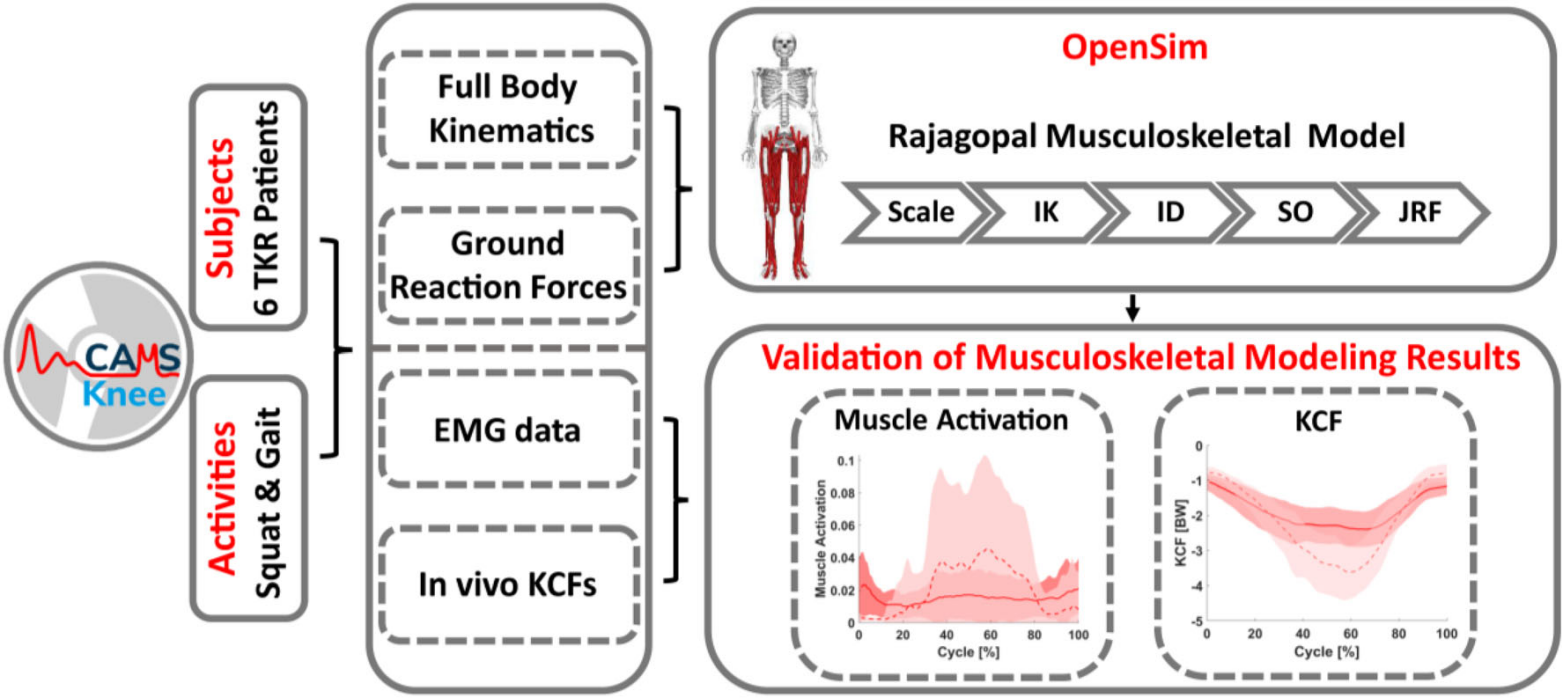
The CAMS-Knee datasets were used to validate musculoskeletal simulation predictions of KCFs and muscle activations for six total knee replacement (TKR) subjects perfoming level walking and squatting. The OpenSim platform was used to scale a generic model,^33^ perform inverse kinematics, inverse dynamics, static optimization, and joint reaction force analysis to calculate the KCFs.

OpenSim (version 3.3) was used to simulate the measured movements and predict KCFs.^8^ A generic full body musculoskeletal model,^33^ with 37 degrees of freedom (DOFs), 80 muscle-tendon units, and 17 torque actuators was used for the simulations. This model included 6 DOFs at the pelvis, 3 DOFs at the hip, 1 DOF at each the knee and ankle. For each subject, the generic model was scaled to match anthropometry based on the positions of skin markers placed over bone landmarks during a static reference trial. Inverse kinematics (IK) was then used to calculate the joint angles, and inverse dynamics (ID) to calculate the intersegmental moments and forces throughout each trial. Then, muscle activations were calculated using the static optimization (SO) tool, which minimized the sum of the muscle activations squared at each time frame.^1^ Finally, a joint reaction force (JRF) analysis was performed to compute KCFs (Fig. 1) in the tibial reference frame. The forces were computed in the tibial reference frame of the musculoskeletal model, which is located approximately at the midpoint of the femoral condyles with the knee in full extension.^2^,^33^ Because the axes of the tibial reference frames of the model and implant measurements are approximately aligned, but the origins have different positions, comparisons were only made between the contact forces, but not the contact moments.

### Data Analysis

The predicted muscle activation patterns were compared against the experimentally measured EMG signals for each trial. The raw EMG signals were bandpass filtered (4th order Butterworth, lowpass 10 Hz, highpass 300 Hz), offset corrected, rectified, and finally low pass filtered with moving average filter (window 0.25 s). Then, the EMG signal of each muscle was normalized to the maximum of muscle activiations value across all trials of both activities for each subject. Because EMG signals are difficult to normalize and subject to measurement error,^16^ only qualitative comparisons were made between the predicted muscle activations and measured EMG signals. Data analysis was performed using MATLAB (R2017b, MathWorks, USA).

To evaluate the accuracy of the simulation predictions, the predicted and measured knee joint contact forces for all trials of each subject over each activity cycle were compared. The root mean square (RMS) error and *R*^2^ Pearson correlation coefficient between the measured and predicted contact forces was computed for each measurement trial and averaged across all subjects for each activity. Additionally, a relative error criterion (${\text{KCF}}_{\text{error}}$) was used to compare the predicted KCF components (${\text{KCF}}_{\text{predicted}}$) against the measured components (${\text{KCF}}_{\text{measured}}$) (Eq. 1).^38^

1 $${\text{KCF}}_{\text{error}} \left[ \% \right] = \left[ {\left( {{\text{KCF}}_{\text{predicted}} - {\text{KCF}}_{\text{measured}} } \right)/ {\text{KCF}}_{\text{measured}} } \right] \times 100$$

## Results

### Knee Contact Forces

**Figure 2:**
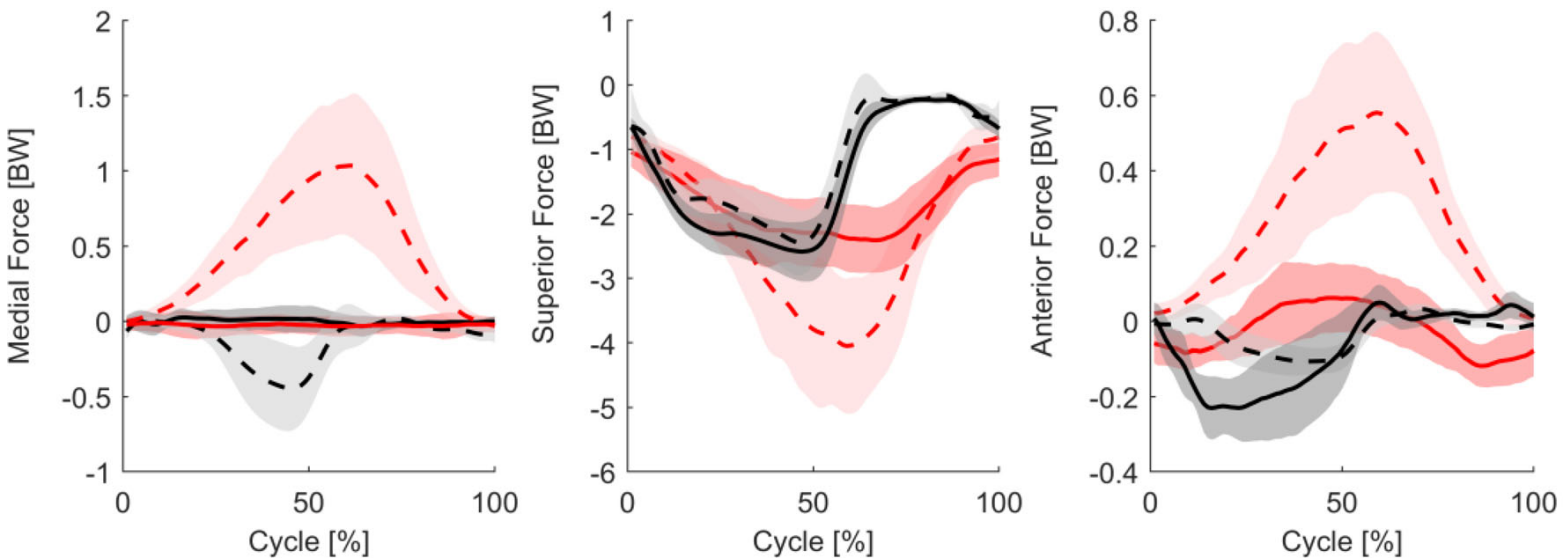
The predicted (dashed) and measured (solid) KCFs for all subjects performing level walking (black) and squatting (red). The bold lines represent the mean across all subjects and all trials, while the shaded areas represent ± 1SD.

The simulations generally underestimated the compressive KCFs during the stance phase of level walking (Fig. 2, middle) but showed good predictions for the swing phase. The only exceptions were for subjects K3R and K8L, where the compressive contact forces were overestimated at the second peak (late stance phase) (Supplementary material Fig. S1). The RMS error between the predicted and measured total contact force throughout the gait cycle averaged across all subjects was 47.5%BW (*R*^2^ = 0.92). The average predicted and measured peak total contact forces were 2.64 BW and 2.36 BW respectively. The anterior contact force was under-predicted throughout the mid stance phase. The lateral component of the contact force was over-predicted during stance phase, coinciding with peak gastrocnemii activation (Fig. 6).

Substantially larger errors in the predictions of the KCF were observed during squatting compared to gait. Near the initial and final standing poses, the superior contact force magnitude was under-predicted (Fig. 2). However, the magnitudes of all three force components were greatly over-predicted during the majority of the activity, especially during deep squat. As a result, a general overestimation of the KCFs was observed over the entire squat cycle, with an average RMS error across all subjects of 105.7%BW (*R*^2^ = 0.81). The peak total contact force occurred at the instant of deepest squat, where the model predicted an average of 4.59 BW, whereas the average measured value was 2.60 BW.

**Figure 3:**
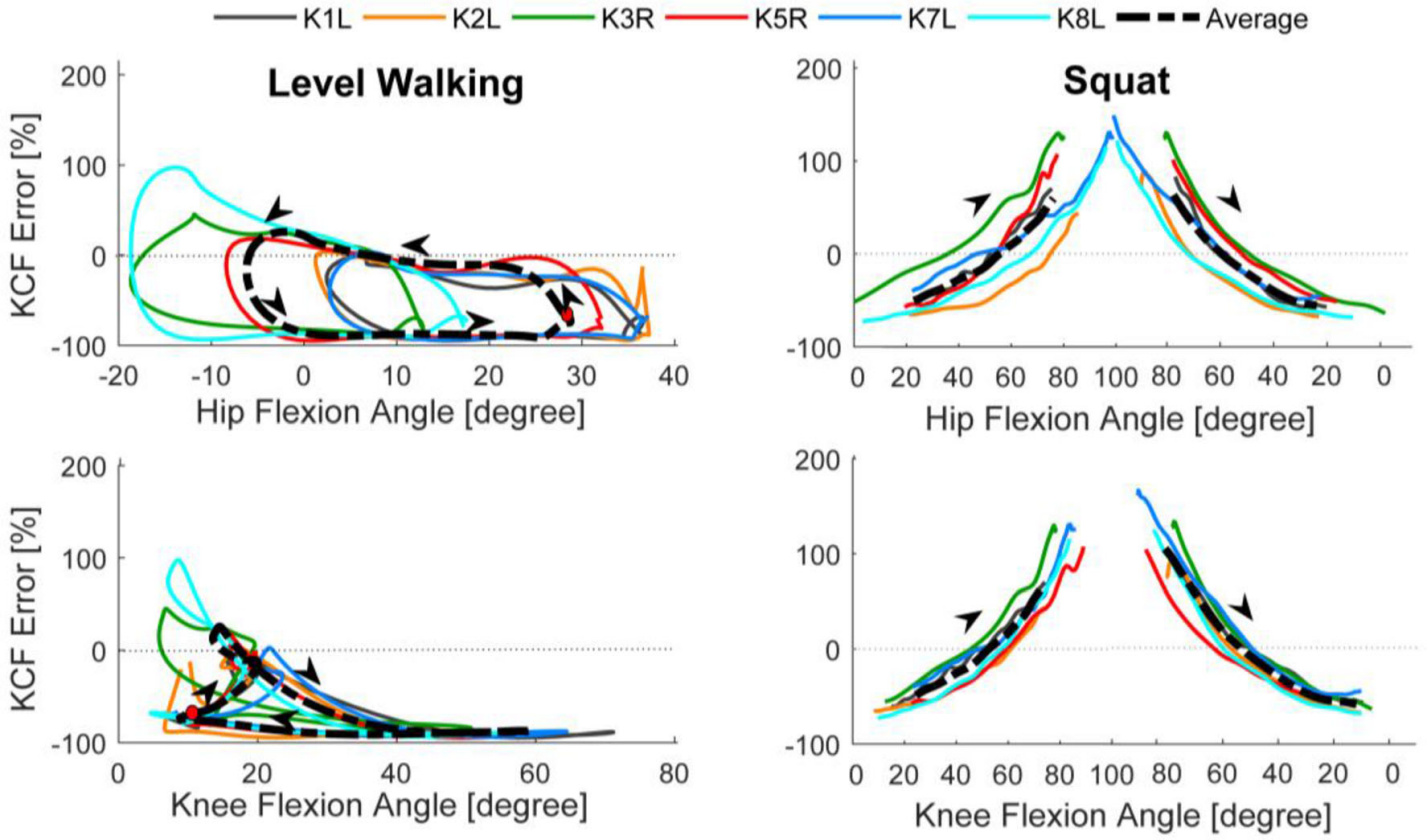
The percent error in the predicted total KCF plotted against the hip and knee angles during level walking and squatting. The bold dashed black line represents the mean of all subjects. The colored lines represent the mean of all trials for each single subject. In the level walking plots, the red circles represent heel strike and the arrows designate the direction of the gait cycle. In the squat plot, the initial standing pose is shown to the left, and the final standing pose to the right.

The relative error in the predicted contact forces showed clear correlation with the hip and knee flexion angles for the squat activity, but no such correlation was observed for level walking (Fig. 3). To clarify the interpretation of this figure, the hip and knee angles throughout both activity cycles are also provided (Supplementary material Fig. S3). During squatting, the model consistently under-predicted the contact forces at extended hip and knee angles, and over-predicted contact forces in deep hip and knee flexion. During level walking, substantial under-prediction was observed over the swing phase (average relative error 84%), with smaller relative errors found during stance phase (average relative error 22%). No trend was observed between hip flexion angle and error in the predicted contact force. At the knee, the relative errors indicated under-predictions were observed at high flexion angles, whereas the relative errors in extended knee postures differed between the stance and swing phases. The relatively high contact force errors were therefore not merely joint angle dependent, since at deep knee flexion angles, the model substantially under-predicted KCFs during walking and over-predicted KCFs during squatting.

**Figure 4:**
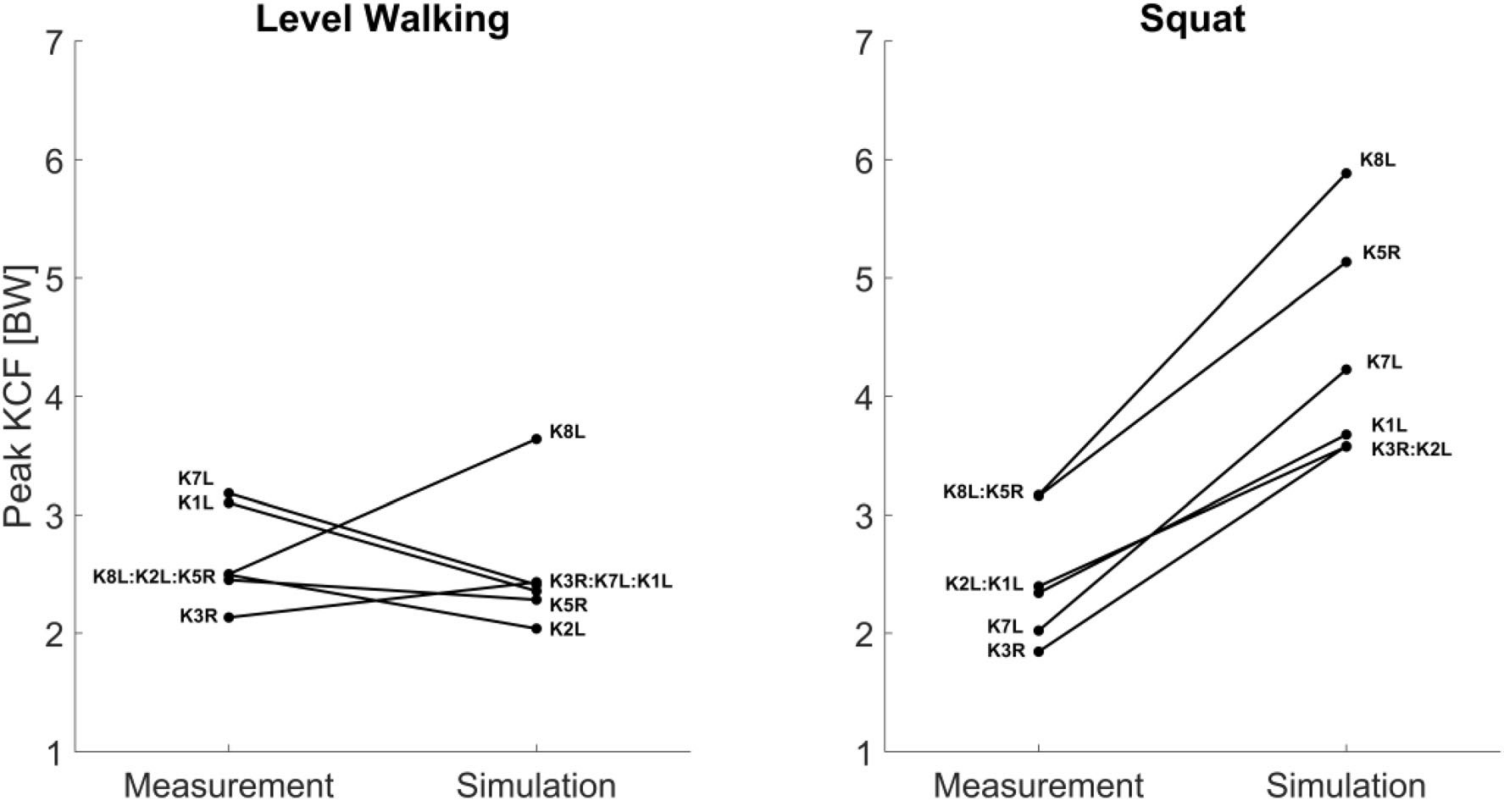
Comparison of the predicted and measured peak KCF for each subject averaged across all trials of walking and squatting.

Interestingly, while the simulation predictions of KCF showed far greater absolute errors for squatting compared to walking, the predicted differences in peak KCF between subjects showed better agreement with the measurements for squatting over walking. A comparison of the subject order in lists based on measured and predicted peak KCFs shows K1L and K2L changed rank for the squat activity (Fig. 4, right). For level walking, the subject rankings based on peak KCF are substaintially different between the measured and predicted list (Fig. 4, left).

**Figure 5:**
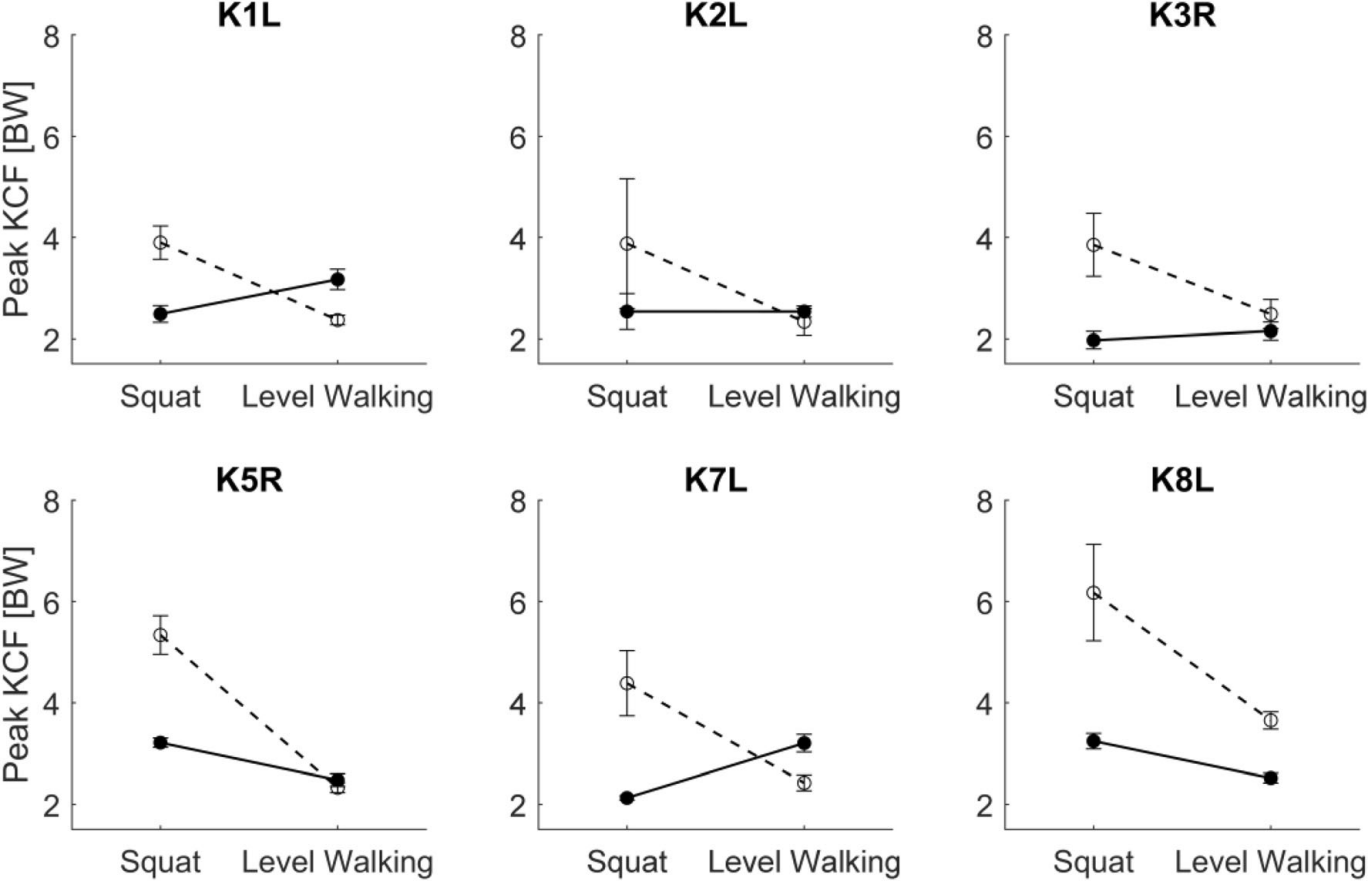
Predicted and measured peak contact forces for level walking and squat for each subject. The open circles and dashed lines represent the mean of the simulations for all trials, the closed circles and solid lines represent the mean of the measurements. The error bars indicate the range of all trials.

The model predictions of the difference in peak contact force between level walking and squatting did not match the measurements (Fig. 5). For all subjects, the simulations predicted that the KCFs during squatting were greater than those during walking. However, the actual measurements indicated that the peak contact forces were generally similar between the two activities, with some subjects showing greater forces during walking while others showed greater forces during squatting.

### Muscle Activations

**Figure 6:**
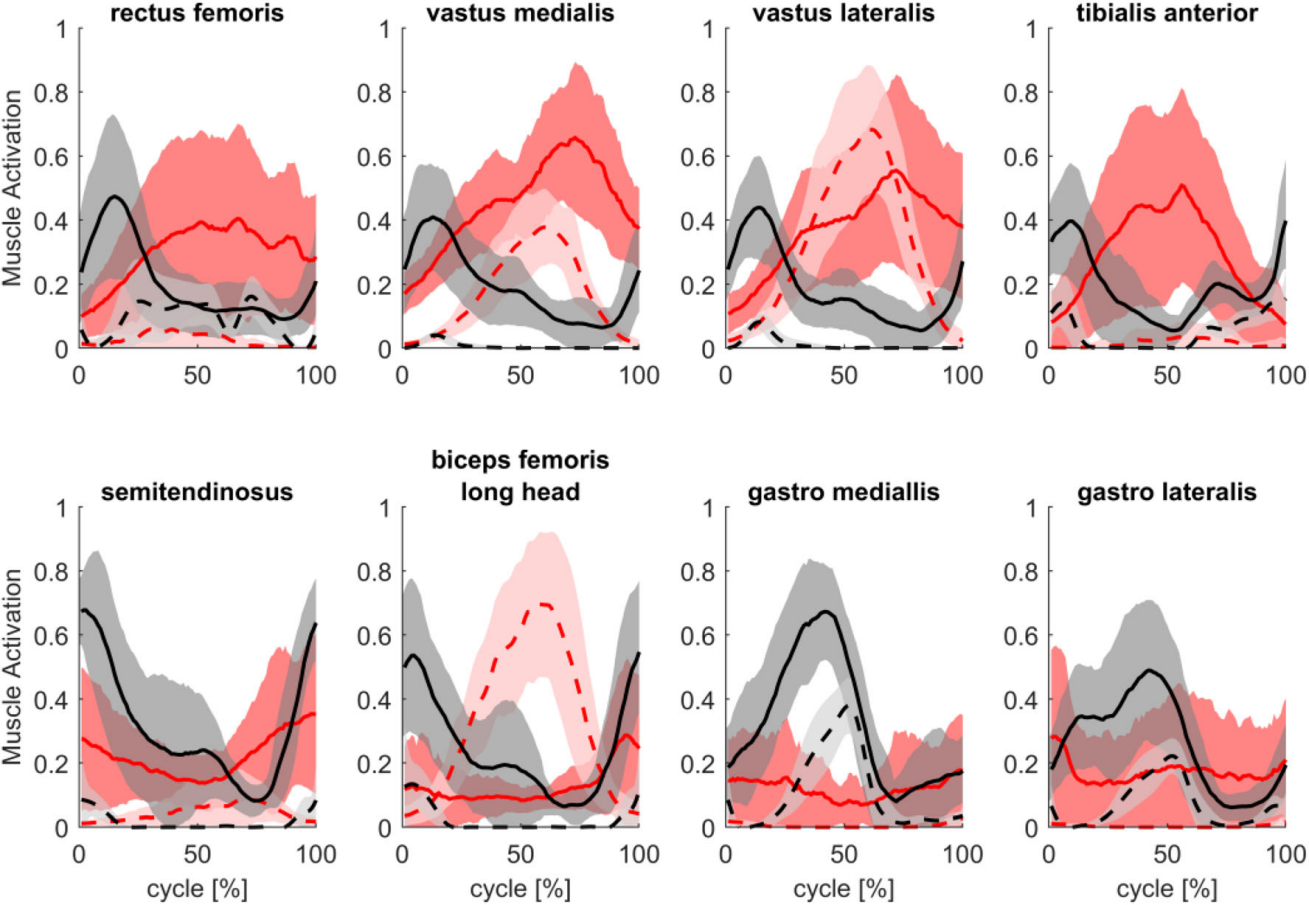
The predicted (dashed) and measured (solid) muscle activity for all subjects performing level walking (black) and squatting (red). The bold lines represent the mean across all subjects and all trials, while the shaded areas represent ± 1SD. The data is presented only for the leg with the instrumented implant.

For level walking, there was generally good agreement between the trends in predicted muscle activations and the EMG measurements for all subjects (Figs. 6, S2). The EMG measures of the vastii showed the characteristic peak during load acceptance, which was also present in the simulation results. Here, the models failed to capture the early peak in the activation of the rectus femoris at load acceptance compared to the measured EMG signals. The hamstrings’ EMG signals and simulated muscle activations increased during terminal swing and peaked just after heel strike. The gastrocnemii EMG signals peaked during push off. A similar pattern was predicted in the simulations, however peak gastrocnemii activity was predicted slightly earlier in the stance phase.

Compared to walking, the predicted muscle activations for squatting showed considerably larger descrepancies from the EMG measurements. The simulated biceps femoris long head activity showed a distinct peak during deep squat, but the measured EMG signal showed minimal activation with only a small peak at the end of the squat cycle. The measured tibialis anterior EMG signal displayed a peak during deep squat, while the simulations predicted minimal activity throughout the whole squat motion. The gastrocnemii were minimally activated in both the simulations and the measurements, while the rectus femoris was predicted to be minimally activated throughout the entire squat motion.

The measured quadriceps EMG signals increased as the subjects lowered into deep squat, and decreased when the subject returned to standing, but a substantially greater activation level was observed between the final and initial standing poses (resulting in an average of + 5.3% in measured KCF). Interestingly here, the hip, knee, and ankle angles and moments all returned to their intial values. Thus, given the measured peak in biceps femoris EMG at the end of squatting, there was likely some residual co-contraction at the end of the squat activity. However, the predicted vastii activation demonstrated a symmetric pattern across the squat cycle, with similar activation levels between the final and initial standing poses. As such, the models were unable to capture the co-contraction observed during the experimental conditions.

## Discussion

Musculoskeletal models allow the non-invasive estimation of muscle and joint contact forces, but previous studies have indicated that substantial errors are present, especially when generic models are used.^19^,^36^,^38^ It is critical that the errors in such models are studied across subjects and activities to establish their applicability and allow an improved understanding the etiology of the errors. This study assessed the accuracy of a widely used musculoskeletal simulation framework (OpenSim) and generic musculoskeletal model,^33^ to predict KCFs and muscle activation patterns during level walking and squatting based on measurements of six patients with instrumented implants.^42^ Generally, the KCFs were under-predicted during the stance phase of level walking, but substiantially over predicted during deep squat. The predicted muscle activation patterns largely agreed with the EMG signals for level walking, but squatting exhibited large discrepencies, especially in the activation patterns of the hamstrings. The models demonstrated limited ability to differentiate between subjects and activities based on a ranking of the peak contact forces. These results demonstrate that predicting subject-specific KCFs using traditional musculoskeletal simulation approaches (scaled generic model and static optimization) remains a considerable challenge and that future simulation study designs must take this uncertainty into account.

Errors in musculoskeletal model predictions of KCFs can generally be attributed to four main factors: (1) errors in the experimentally derived joint kinematics and external forces,^23^ (2) inaccurate representation of the anatomy and physiology of the musculoskeletal system, (3) uncertainty in subject-specific model parameters, and (4) an incomplete understanding of the solution to muscle redundancy. Inaccuracy in the representation of the musculoskeletal system includes the basic model of muscle contraction that ignores history dependencies,^28^ simplified representation of the three-dimensional muscle fiber paths,^5^ and idealized kinematic joints that do not include contact or passive soft tissue structures.^22^,^50^ The personalization of musculoskeletal models remains a major challenge, and errors in joint centers, muscle moment arms, and muscle-tendon model parameters can have a significant effect on predicted contact forces.^27^,^30^,^46^,^50^ Finally, the ability to solve muscle redundancy remains a considerable challenge,^15^ and different optimization criteria can yield substantially different predictions of muscle activation patterns and consequently KCFs.^10^,^39^ Inherently, our simplistic approach of scaling a generic model and performing static optimization contains errors due to each of these factors. While the sensitivity of model predictions to many of these factors have been evaluated in different combinations,^10^,^30^,^46^ the relative importance of each factor to accurately predict KCFs requires more extensive study.

In light of these known limitations, we aimed to better understand the observed clear discrepancies in predicted versus measured KCFs during squatting, especially considering the relatively good results achieved during walking. The substantial error in the KCF predictions during deep squat (Fig. 2) was likely caused by a disproportionate co-contraction of the hamstrings and quadriceps. Here, the simulations predicted excessive activation of the biceps femoris long head muscle compared to the EMG signals. To balance the external moments observed during deep squat, the model must generate large knee extension moments, hence necessitating activity of the quadriceps. In addition, to balance the hip flexion, internal rotation, and abduction moments, activation of the hip musculature is required. Here, the combination of the optimization cost function, muscle-tendon parameters, and muscle moment arms led the hamstrings (specifically the biceps femoris long head: see Fig. 6) to be activated to generate the hip moments. Due to their biarticular function, the predicted hamstrings forces resulted in additional knee flexion moments, which must be overcome by further quadriceps activation. This co-contraction at the knee leads to increased contact force predictions, which can explain the large errors observed during squatting that are not seen during walking. Unfortunately, direct measurement of gluteal activity to verify these assumptions was not taken within the CAMS-Knee measurements. As a result, while the evidence provided in this study is compelling, further investigation into this overloading mechanism through exaggerated co-contration is clearly required in other datasets.

Possible improvements to the model to reduce the hamstrings activation include a more refined representation of the muscles crossing the hip to improve the capacity of the gluteus muscles to generate hip flexion moments at deeper angles, and improve representations of the abductor muscles to generate the hip internal rotation and abduction moments. Two studies have proposed adapting the muscle paths or modifying the wrapping surfaces of the Rajagopal model to improve the ability to simulate activities with high knee and hip flexion.^6^,^21^ Both of these studies exhibited improvements in the predicted EMG patterns during pedalling and squatting activities. However, for squatting the updated model still showed high activation of the biceps femoris long head during deep squat,^6^ similar to the results observed in our study, suggesting that further improvement is still required. In future, it will be important to benchmark such model adaptations using the CAMS-Knee datasets to investigate whether they improve predictions of KCFs.

The limited capacity of the model to correctly predict the relative ranking of the subjects and activities based on peak KCFs is an important finding (Figs. 4 and 5). Musculoskeletal simulations are commonly applied to estimate joint contact forces in groups of healthy and pathologic subjects and to investigate the role of joint loading in pathologies.^4^,^20^,^29^,^34^,^35^,^40^,^47^,^49^ However, musculoskeletal models have traditionally overestimated the absolute magnitude of joint loading,^41^,^43^ our results indicate that the capacity of traditional musculoskeletal modeling techniques to relatively differentitate the order of knee joint loading between subjects is also limited. Advances in modeling techniques such as the inclusion of EMG driven simulations,^17^ or detailed six DOF knee models,^29^ may improve the capacity of musculoskeletal simulations perform such studies. The CAMS-Knee datasets will provide an important resource for benchmarking these novel techniques and evaluating their ability to predict KCFs.

While many studies have validated musculoskeletal model predictions of KCFs during walking,^19^ other activities of daily living are less well investigated. One study found average peak KCF errors of 11% for level walking, 26% for stair climbing, 15% for sit-to-stand and 14% for squat.^43^ Our original study also used the CAMS-knee data set, but a different musculoskeletal model (Gait 2392), and despite updating the modelling tools used in this study, we have found similarly high average peak force errors for squat (110%) to those observed using the original model (59%).^38^ Here, our results were highly subject specific, with an average peak force error of about 60% for some subjects (e.g., K2L), but more than 140% for others (e.g., K7L). This study builds upon our original study by providing an understanding of the etiology of the underlying modelling errors through comparison of the muscle activation predictions against EMG. The next stages of this process require an adaption of the musculoskeletal models to e.g., improve lever arms of the gluteal muscles *etc*,^32^ and investigate whether the reported errors can be mitigated. This continuing process highlights the importance of benchmarking musculoskeletal modeling techniques using multiple subjects and activities.^43^

This study provides a demonstration of the comprehensive validation of musculoskeletal model predictions enabled through the CAMS-Knee datasets. However, the datasets are still limited to six elderly patients with total knee replacements. Thus, it remains unknown whether the presented limitations of musculoskeletal model predictions can be extrapolated to healthy subjects or patients with pathologic knee conditions. Furthermore, we used a publically available musculoskeletal model,^33^ and simulation software,^8^ to perform static optimization and predict KCFs. However, other models and simulation algorithms will likely result in different KCF predictions. Thus, our results simply express the likely lower bound of accuracy that can be achieved in KCFs predictions using musculoskeletal models. Finally, we only personalized the musculoskeletal models by linear scaling based on a static motion capture collection, further personalization that accounts for subject specific musculoskeletal geometries and component alignment may reduce errors in KCF predictions. Despite these limitations, our average errors in the predicted peak forces for level walking were only 22%, thus demonstrating the efficacy of this gait model for investigating walking activities. This study therefore provides an initial benchmark using the CAMS-Knee datasets of walking and squatting, and demonstrates the importance of validation for all future simulation approaches.

Several guidelines have been proposed for evaluating musculoskeletal model predictions,^16^,^26^ that define *in vivo* joint contact force measurements as the ‘gold standard’ for validation. The CAMS-Knee datasets will now compliment the Grand Challenge to Predict In Vivo Knee Loads, as publicly available validation benchmark datasets. This study intended to provide a baseline assessment of a scaled generic model,^33^ and open-source OpenSim static optimization tool.^8^ As more complex modeling methods and simulation routines are developed, their accuracy in predicting subject-specific knee loads can be compared against this traditional approach. However, the uncertainty in measuring patient-specific model parameters and muscle redundancy remain major obstacles in the accurate prediction of patient-specific knee loading.

In this study, we demonstrate that current generic musculoskeletal modelling techniques are able to reproduce the *in vivo* conditions measured during walking. However, large errors were observed in loading predictions during activities that involve deep flexion, and we present compelling evidence that these limitations lie in the activation patterns of the hip musculature. Importantly, in addition to the observed errors in the absolute magnitude of the predicted joint loading, our results indicate that the ability of musculoskeletal models to predict the differences in KCFs between subjects and activities is also limited.

## Electronic supplementary material

Below is the link to the electronic supplementary material.

Supplementary material 1 (DOCX 41990 kb)
