# Supplementary material for: The Capacity of Generic Musculoskeletal Simulations to Predict Knee Joint Loading Using the CAMS-Knee Datasets
Source: Ann Biomed Eng. 2020 Jan 30;48(4):1430–40. doi: 10.1007/s10439-020-02465-5 (PMC7089909; doi:10.1007/s10439-020-02465-5)
Supplement: Supplementary file 1 — Supplementary material 1 (DOCX 41990 kb) [file 10439_2020_2465_MOESM1_ESM.docx]

**Supplemental Material**

**Subject-Specific Knee Contact Load Validation**

**Subject K1L**


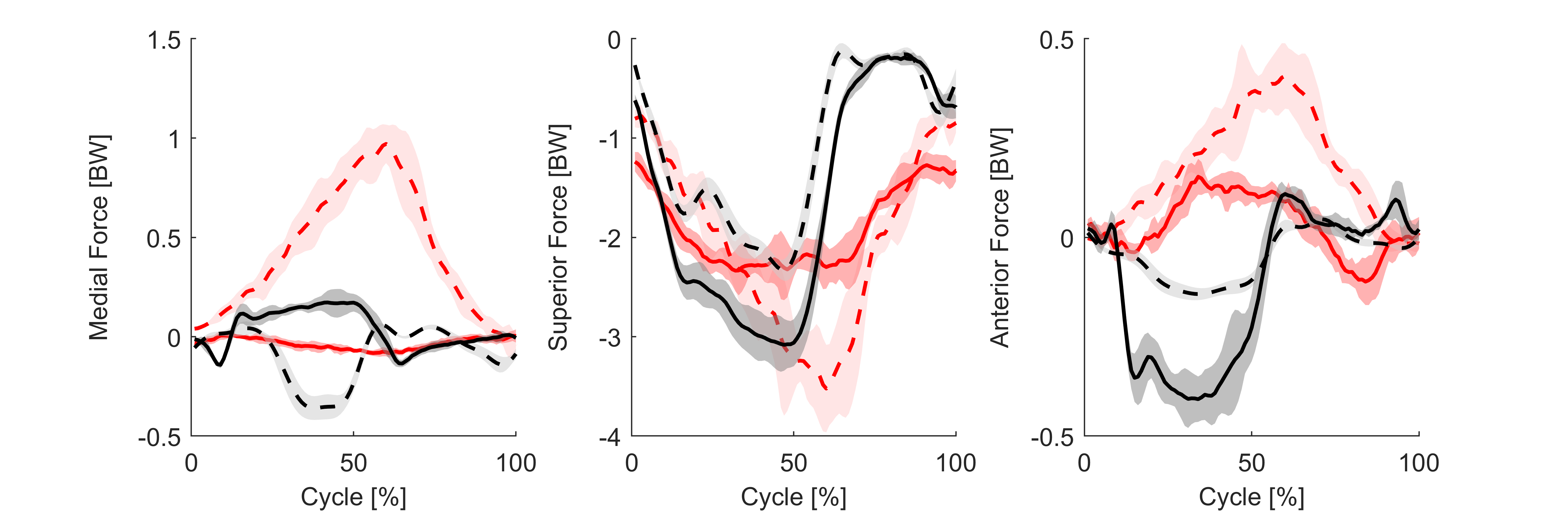


(a)

**Subject K2L**

**
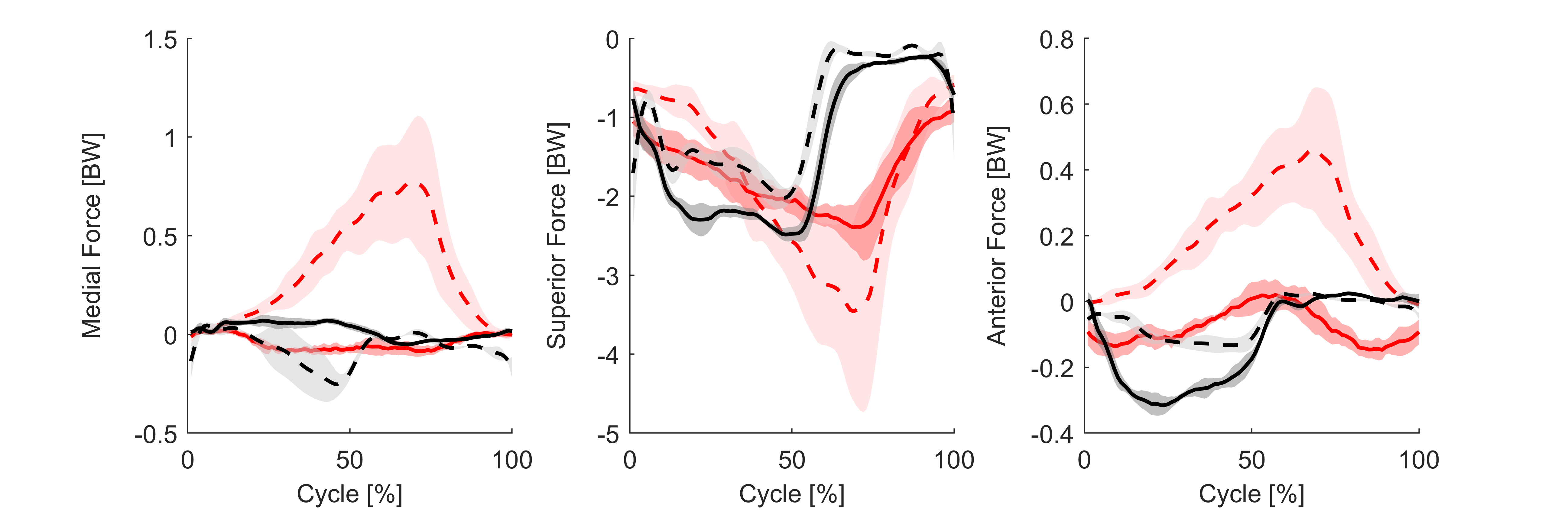
**

(b)

**Subject K3R**

**
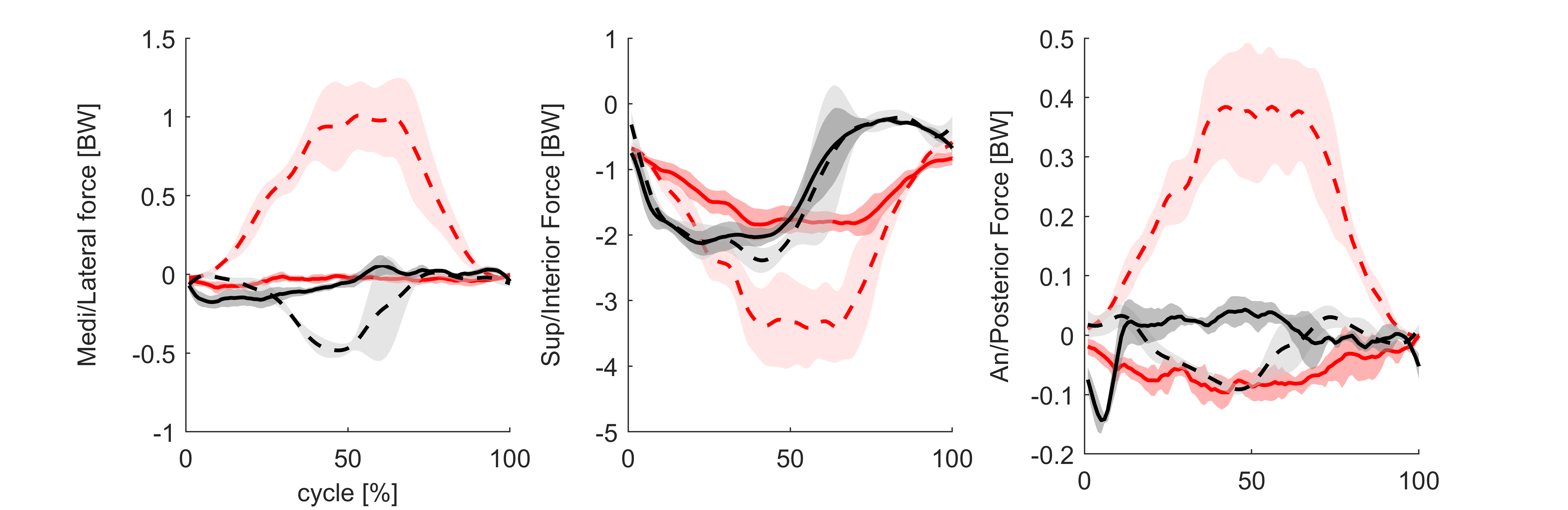
**

(c)

**Subject K5R**

**
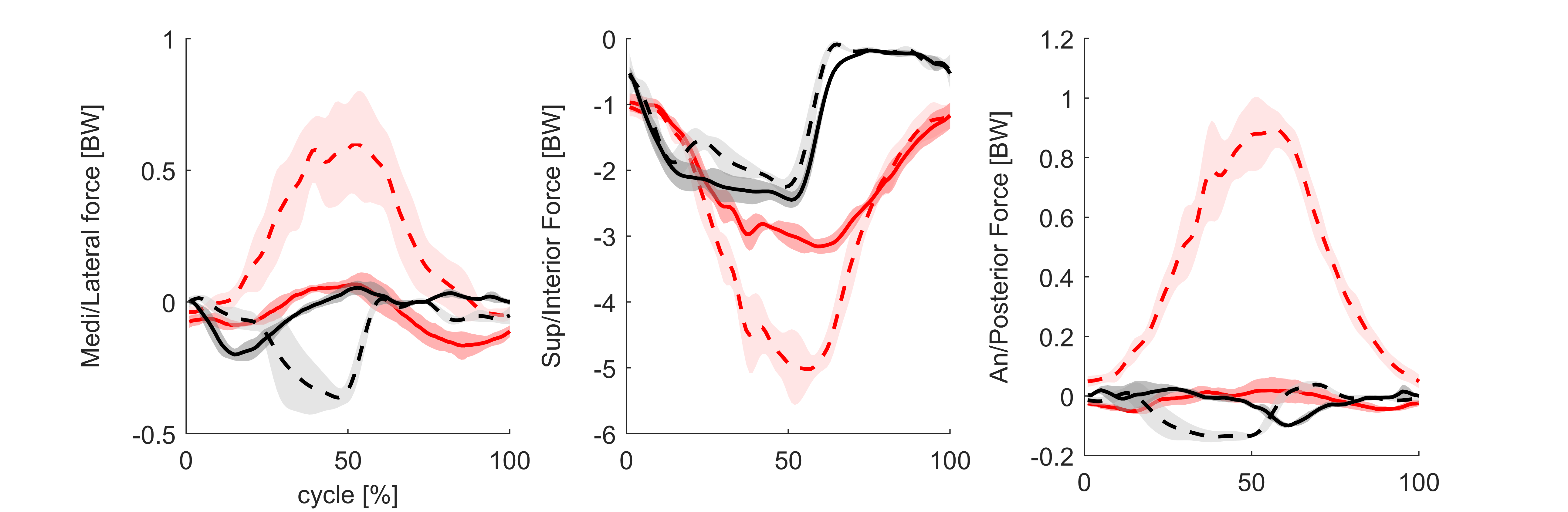
**

(d)

**Subject K7L**

**
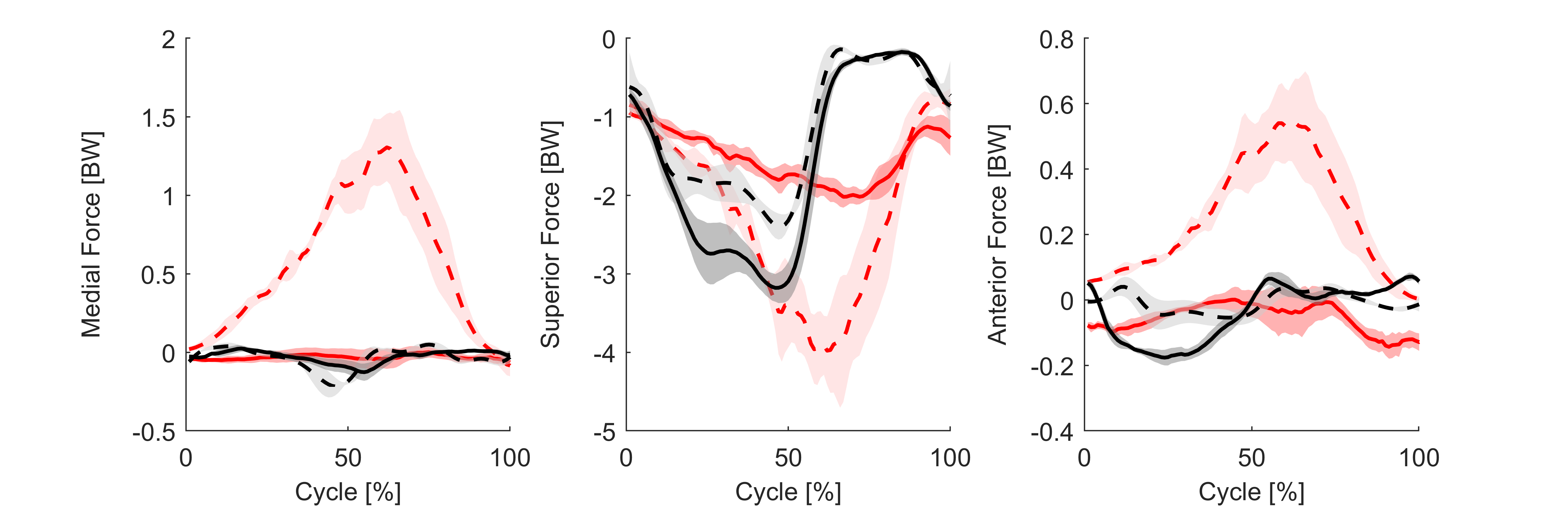
**

(e)

**Subject K8L**

**
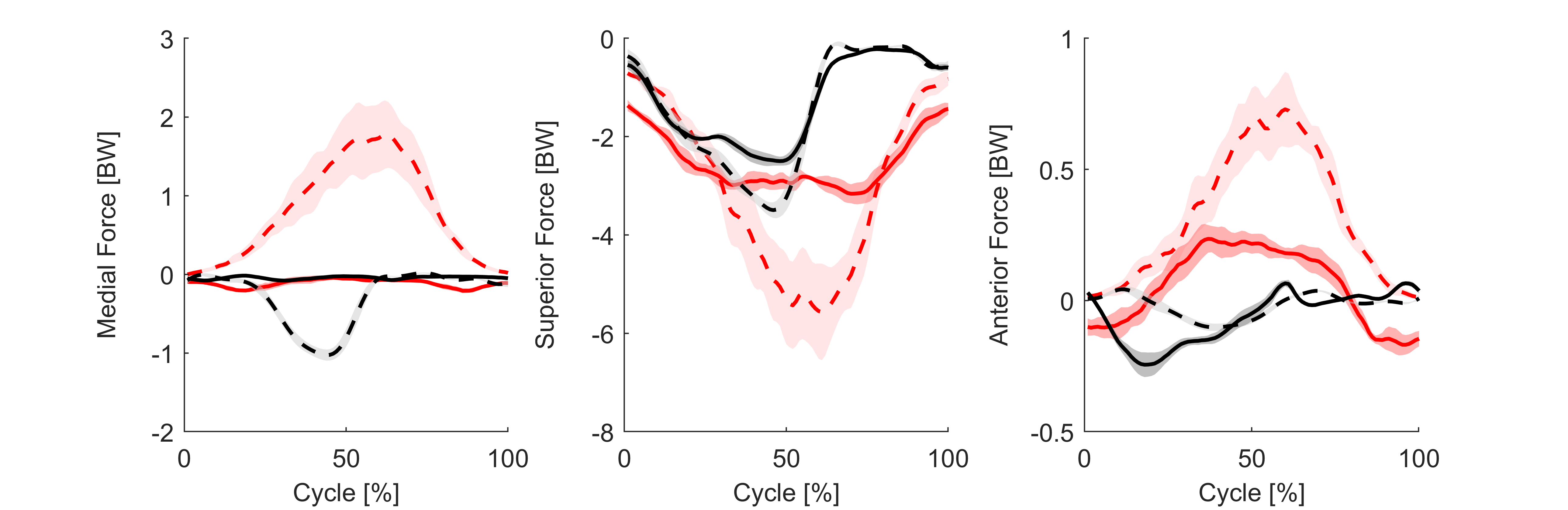
**

(f)

*Figure S1: The predicted (dashed) and measured (solid) KCFs and for each subject performing level walking (black) and squatting (red). The bold lines represent the mean across all trials, while the shaded areas represent ±1SD.*

**Subject-Specific Muscle Activity Validation**

**Subject K1L**


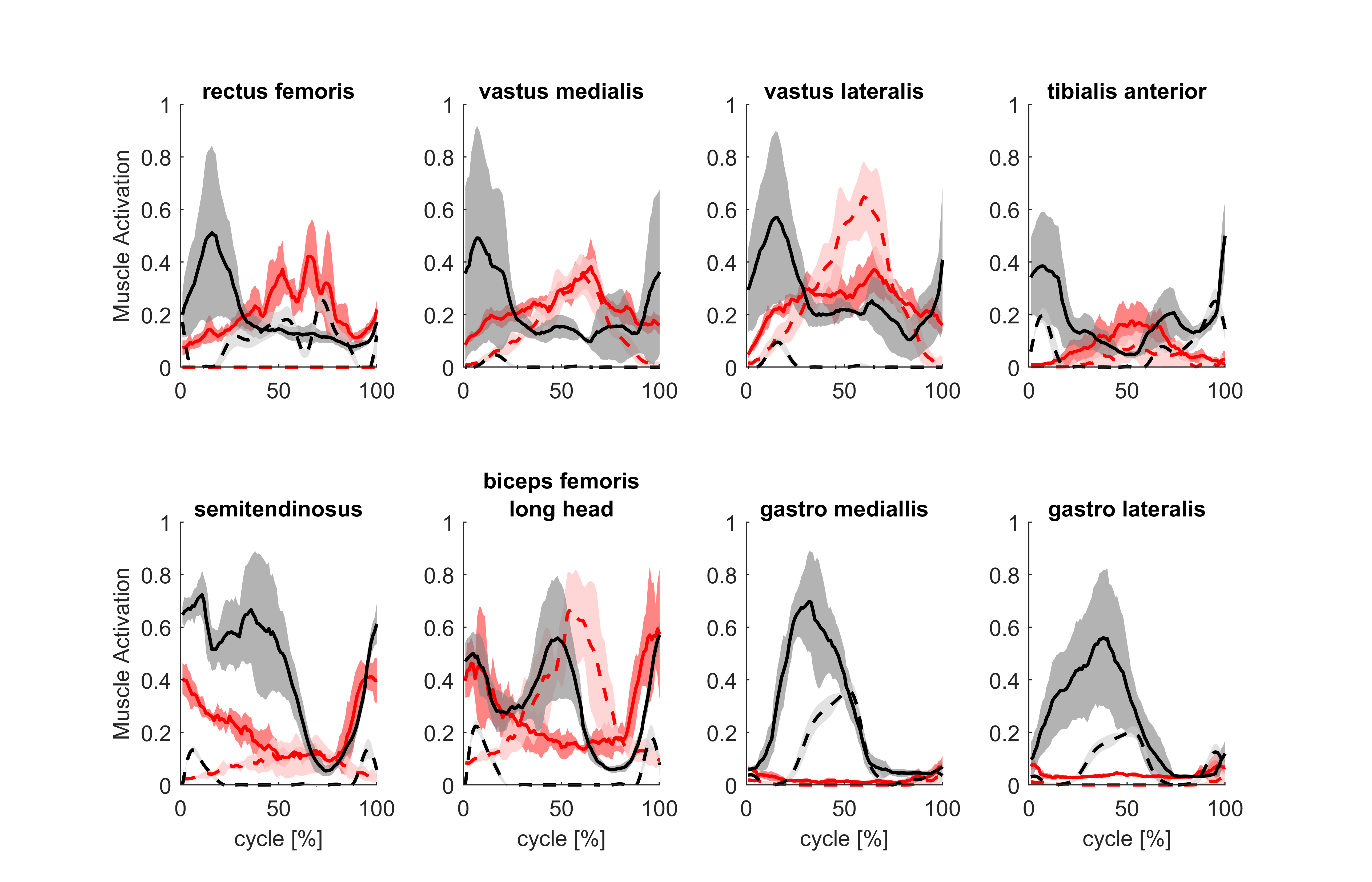


(a)

**Subject K2L**

**
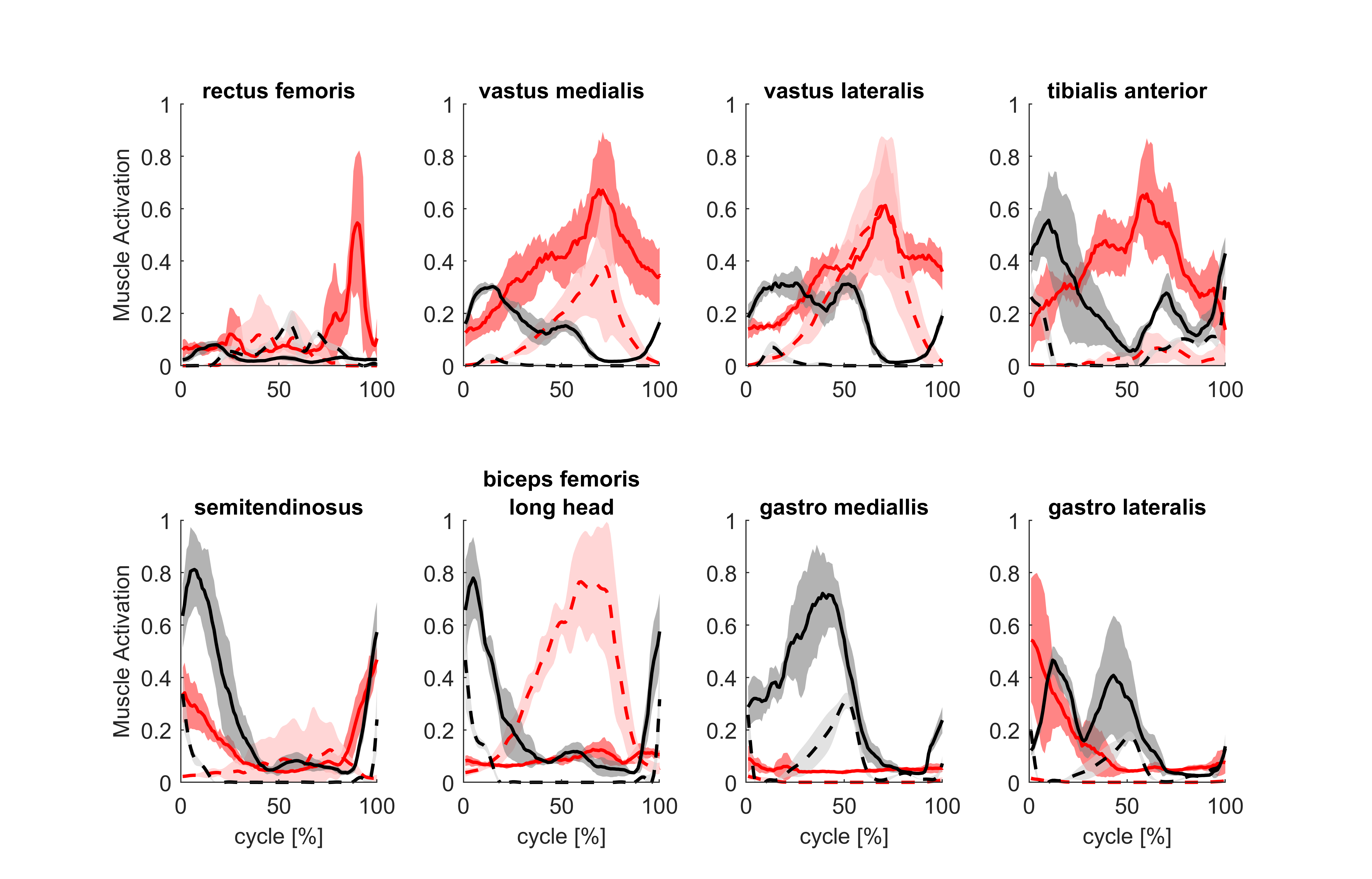
**

(b)

**Subject K3R**

**
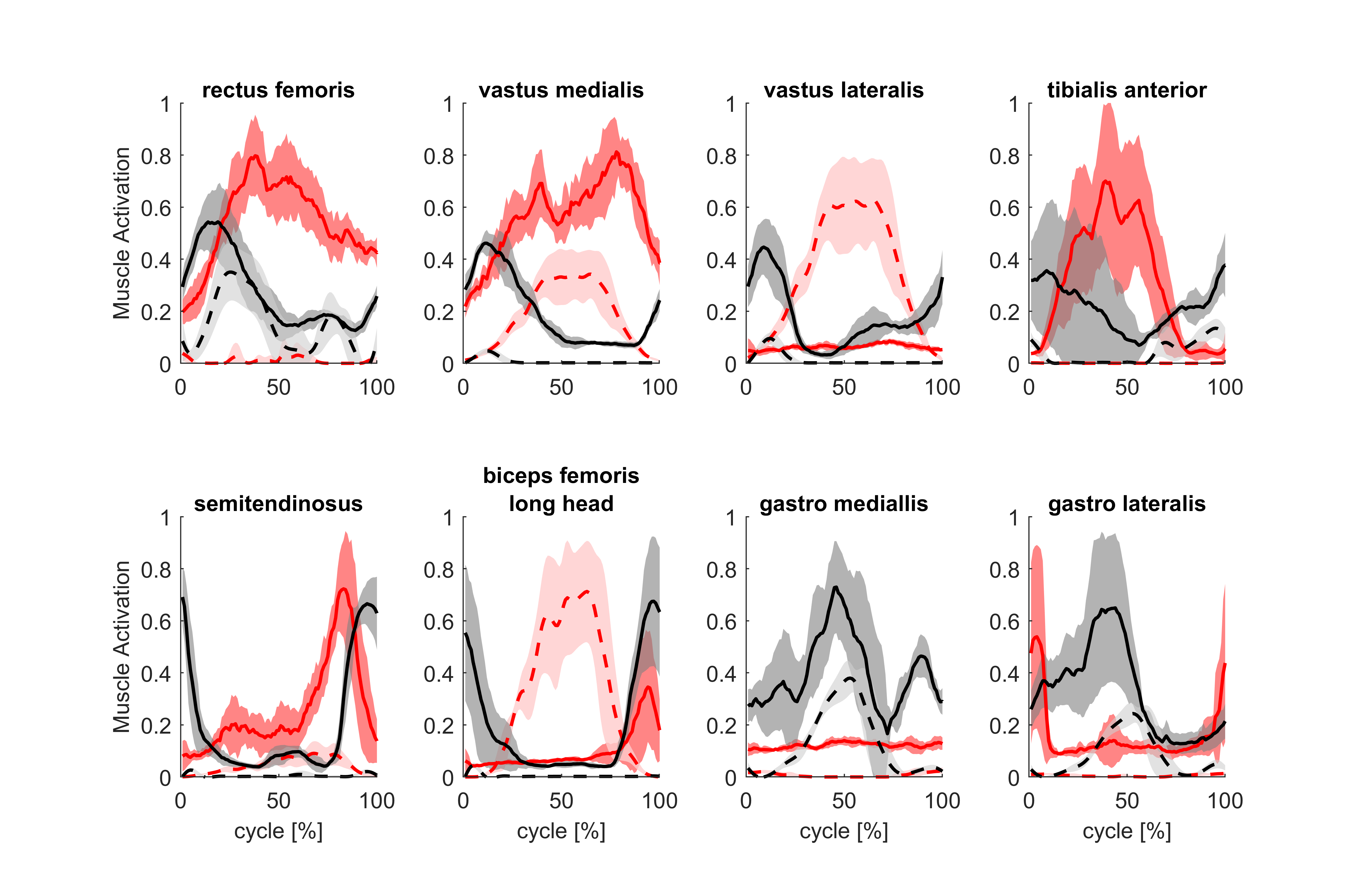
**

(c)

**Subject K5R**


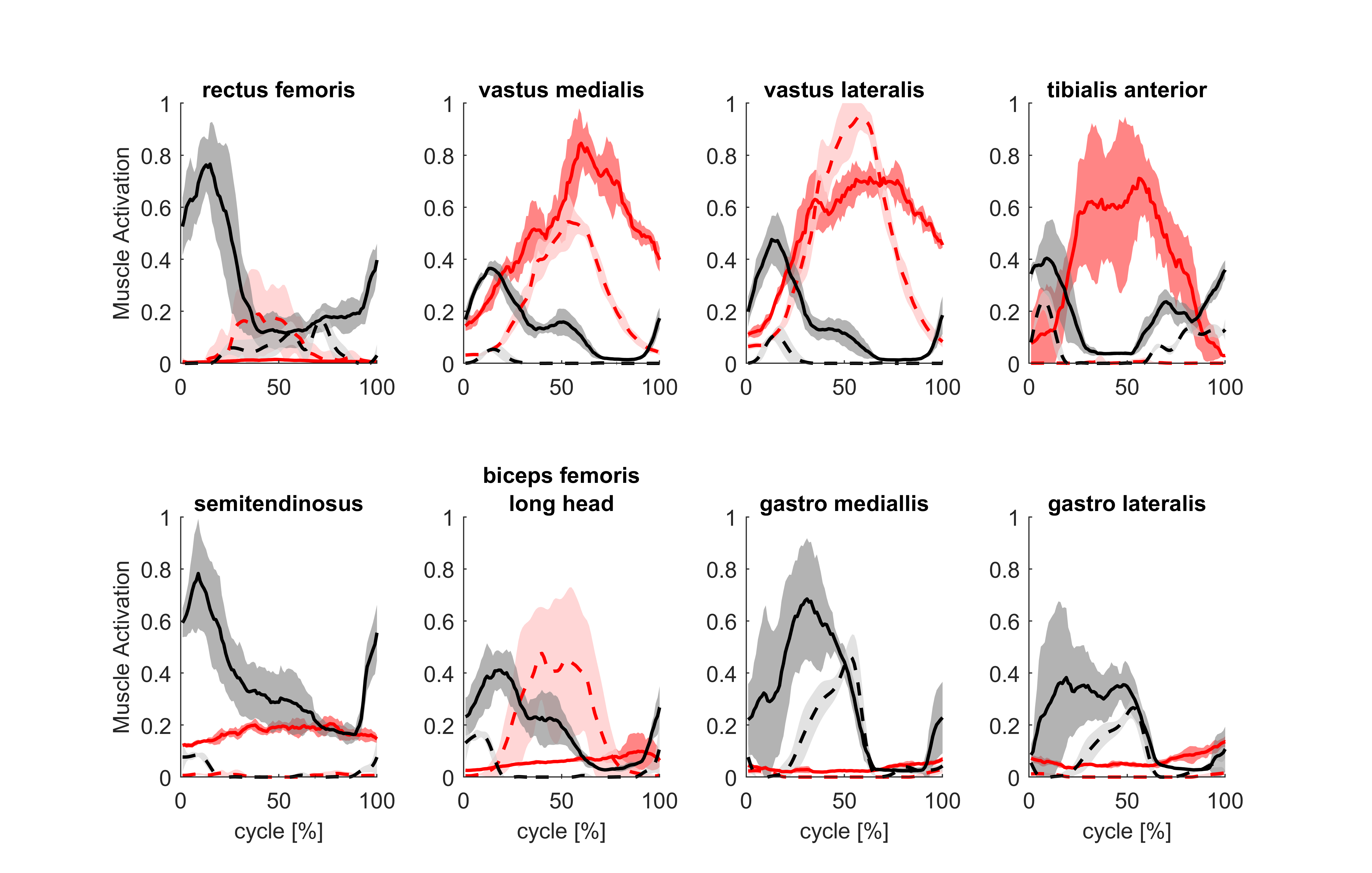


(d)

**Subject K7L**

**
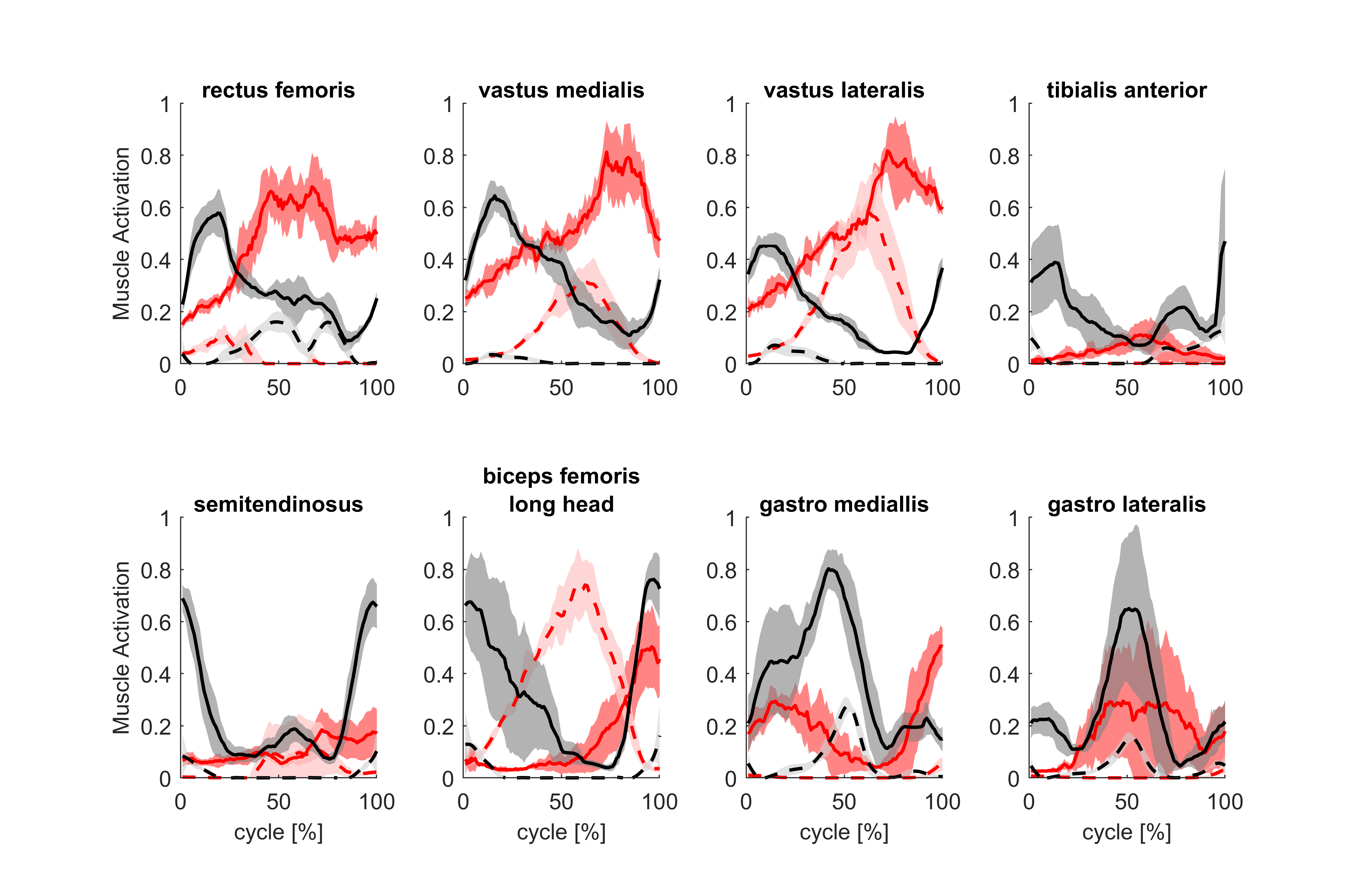
**

(e)

**Subject K8L**

**
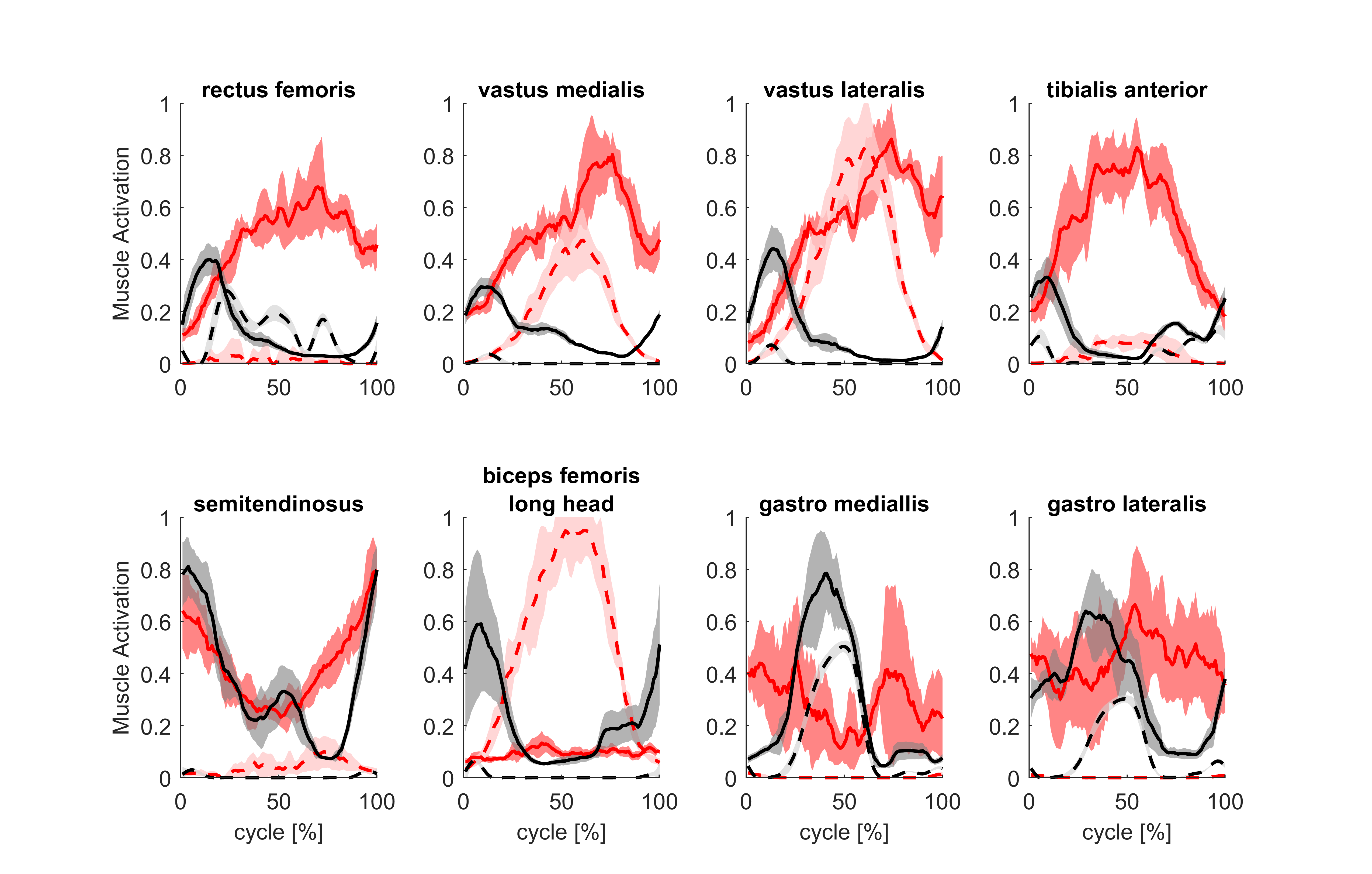
**

(f)

*Figure S2: The predicted (dashed) and measured (solid) muscle activity for each subject performing level walking (black) and squatting (red). The bold lines represent the mean across all trials, while the shaded areas represent ±1SD. The data is presented only for the leg with the instrumented implant.*


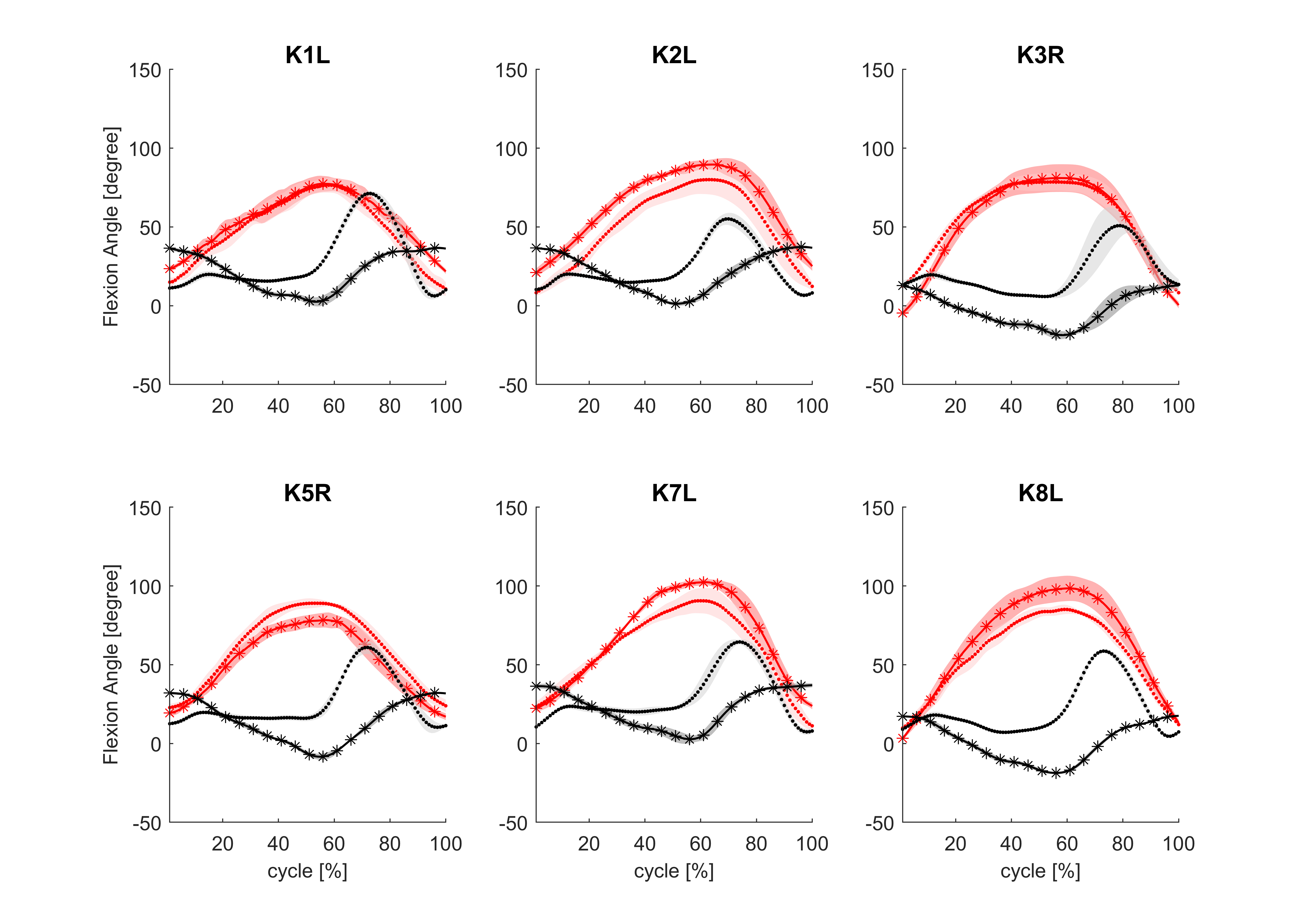


*Figure S3: Knee (circles) and hip (stars) flexon angle for each subject performing level walking (black) and squatting (red). The bold lines represent the mean across all trials, while the shaded areas represent ±1SD.*
